# Use of e-learning in clinical clerkships: effects on acquisition of dermatological knowledge and learning processes

**DOI:** 10.5116/ijme.5a47.8ab0

**Published:** 2018-01-17

**Authors:** Frederike Fransen, Herm Martens, Ivo Nagtzaam, Sylvia Heeneman

**Affiliations:** 1 Department of Dermatology, Maastricht University Medical Centre, Maastricht, The Netherlands; 2 Department of Pathology, School of Health Profession Education, Maastricht University, Maastricht, The Netherlands

**Keywords:** E-learning, mixed methods, dermatology, learning theory, students’ perceptions, clinical phase

## Abstract

**Objectives:**

To obtain a deeper understanding of how the e-learning program, Education in Dermatology (ED), affects the acquisition of dermatological knowledge and the underlying learning processes of medical students in their clinical phase.

**Methods:**

The study used a mixed method design with a convergent parallel collection of data. Medical students (n=62) from Maastricht University (The Netherlands) were randomized to either a conventional teaching group (control group n=30) or conventional teaching plus the e-learning program (application on smartphone) group (e-learning group n=32). Pre- and post-intervention knowledge test results were analysed using an independent t-test. Individual semi-structured interviews (n=9) were conducted and verbatim-transcribed recordings were analysed using King’s template analysis.

**Results:**

The e-learning program positively influenced students’ level of knowledge and their process of learning. A significant difference was found in the post-test scores for the control group (M=51.4, SD=6.43) and the e-learning group (M=73.09, SD=5.12); t(60)=-14.75, p<0.000). Interview data showed that the e-learning program stimulated students’ learning as the application promoted the identification and recognition of skin disorders, the use of references, creation of documents and sharing information with colleagues.

**Conclusions:**

This study demonstrated that use of the e-learning program led to a significant improvement in basic dermatological knowledge. The underlying learning processes indicated that e-learning programs in dermatology filled a vital gap in the understanding of clinical reasoning in dermatology. These results might be useful when developing (clinical) teaching formats with a special focus on visual disciplines.

## Introduction

In today’s general medical practice, physicians are frequently faced with dermatological cases.^1^^,^^2^ However, the limited dermatological training in medical schools stands in stark contrast with the high number of skin diseases that are estimated to be encountered in primary care.^3^

Medical students are insufficiently exposed to dermatological cases prior to their clerkships. Published studies have reported that the time devoted to dermatology in the medical undergraduate curriculum is only 0.24-0.30% of the period of their study, and call for either more or other formats of teaching. This lack of education often results in knowledge gaps and low confidence levels in performing skin examinations and managing cutaneous disorders in clinical practice.^3^^-^^6^ E-learning refers to the use of internet technologies to provide strategies for enhancing knowledge and performance.^7^^,^^8^^,^^9^ The rapid advances in online medical, educational systems have demonstrated promising benefits for learning processes, such as the ability to approach study materials at any time and in off-site locations, the possibility of controlling the depth of content delivery, and the possibility of giving and receiving feedback regularly.^7^^,^^9^^,^^10^ In the field of dermatology, this new teaching concept has shown to increase medical students exposure to skin diseases, which helps build their recognition of common dermatological conditions.^3^  Previous studies^1^^,^^3^^,^^7^^,^^9^^,^^11^^,^^12^ that evaluated e-learning teaching formats in the field of dermatology have stated that students valued the visual and interactive aspects. However, there is still a need to better understand students’ perceptions on e-learning and how it can drive learning. Silva and colleagues^3^ reported that the use of an e-learning program, in combination with traditional teaching methods, resulted in improved retention of knowledge of dermatological topics. They emphasized the need for further exploration, since e-learning may enhance the learning process, and potentially improve the overall learning experience of medical students when studying dermatology. Wahlgren and colleagues^1^ have pointed out that most of the web-based programs in dermatology have been developed for medical students within the preclinical curriculum. However, the value and benefits of e-learning formats for dermatology within the context of clinical clerkships are not yet known.^1^^,^^3^^,^^7^^,^^13^

This study explores the learning benefits of an e-learning program on smartphones among medical students in the clinical clerkships. The objective of this study is to investigate the effect of the e-learning program on the acquisition of dermatological knowledge in comparison to non-program users. The second objective is to provide a deeper understanding of how the e-learning program and its learning benefits are perceived by medical students, and how it can drive learning.

To the best of our knowledge, this study is the first to use a mixed method design to merge quantitative data on knowledge acquisition with qualitative data on students’ perceptions of an e-learning program in dermatology. In addition, this study focuses on students in their clinical clerkships, as opposed to a pre-clinical learning environment.

## Methods

### Study design and participants

The study used a mixed method design with a convergent parallel collection of data in order to create a synergistic understanding of the learning process provided by the e-learning program. The mixed method allowed us to explore to what extent the qualitative data (interviews) complemented the quantitative data (knowledge test scores).^14^

Participants were fourth-year medical students at Maastricht University that were enrolled in a clinical clerkship. During the fourth year, internal medicine is part of the first clerkship offered to medical students. The clerkships take place in the University Hospital of Maastricht or affiliated hospitals in the region. The clerkship in internal medicine consists of 12 weeks, in which students are placed in various internal medicine disciplines such as cardiology, pulmonology, or dermatology for 3-6 weeks. These placements are random; therefore only 2 out of 15 students are assigned to the dermatology ward per round of 12 weeks.

Participants were randomized to either a conventional teaching group (control group n=30) or conventional teaching plus the e-learning program (application on smartphone) group (e-learning group n=32). The conventional teaching consisted of one lecture addressing dermatological topics. The duration of the follow-up period of all participants was six weeks. As each group of students started their first clerkship in different periods, the timeframe of data collection ranged from November 2016 until February 2017.

Participants in the e-learning group were given short verbal and practical instructions on how to use the e-learning program. This program, named Education in Dermatology (ED), is an interactive web-based program that can be accessed from any computer or smartphone. The program contains 35 clinical cases, featuring the most common dermatological diseases. Each case consists of two or three multiple-choice questions regarding the description, diagnosis, and management of the disease. Answers are provided along with examples of typical visual features necessary to evaluate skin lesions. In addition, links and references to learning materials, such as websites, are provided for in-depth information.

The study was approved by the ethical review board of the Dutch Society for Medical Education. Informed consent forms were obtained from all participants prior to the study. Participation in the study was voluntary. Results did not affect students’ grades and were used for research purposes only.

### Data collection methods

#### Quantitative data

The department of dermatology at the University Hospital of Maastricht developed the multiple-choice questions used in the program and the tests. These questions are part of an existing, validated question bank that is used for summative assessment during the clerkships at the teaching hospital of Maastricht University. To ensure reliability, dermatologists and course instructors (HM, IN) critically reviewed the content before using the questions in the pre- and post-tests and e-learning program. Questions were based on the learning objectives and outcomes of the medical curriculum. Each case consisted of three questions that measured different levels of learning: knowledge, combined comprehension and application, and problem-solving ability. Before the start of the intervention, all students were asked to complete the pre-test. After six weeks, students were asked to complete the post-test. Each test consisted of 45 questions. A time limit of 45 minutes was applied. Scores ranged from 0% to a maximum of 100%. To prevent students in the e-learning group sharing information with the control group, individual passwords for the program were provided to students within the e-learning group.

#### Qualitative data

Individual interviews were used to explore students’ experiences of the e-learning program, and their learning approaches. The interview consisted of semi-structured questions based on basic concepts of learning, self-regulation of learning, and user-friendliness of the program (Appendix).^15^^-^^18^ Students of the e-learning group who had fully completed six weeks of study were invited to take part. A total of 9 students participated in the individual interviews, with 4 students using the e-learning during a placement in the dermatology ward and 5 using the e-learning during a placement in a different internal medicine discipline. Interviews were conducted between December 2016 and February 2017 by the first author (FF). Interview time was fifty-sixty minutes. All interviews were audiotaped and transcribed verbatim. We present the results of the interviews through summaries and quotes.

### Data analysis

#### Quantitative data

We compared the pre- and post-test in the e-learning and control group using an independent paired t-test. A p value of < 0.05 was considered statistically significant. The Statistical Package for Social Sciences (SPSS version 21) was used for the data analysis.

#### Qualitative data

Interviews were analysed using a type of theory-based thematic analysis: template analysis. During this exploration, a sequence of coding patterns, involving hierarchically organized themes, was applied to the data.^19^

The template analysis started with a set of predefined codes, based on previous studies in e-learning which also supported the development of the interview questions.^13^^,^^15^^-^^18^Independent analysis of interviews 1–5 (FF and SH) using the predefined codes resulted in an initial template, which was then used in interviews 6-9. This led to the final template that was discussed with the research team (FF, SH, and HM), as represented in Table 2. Following interview 9, all new data fitted in categories already devised, no new insights were obtained, no new themes identified, and no new issues arose regarding a category of data. We, therefore, conclude that data saturation was reached at this point.

To certify that no significant information had been overlooked, all interviews were reread by SH and FF, after which the final template could be confirmed. Thereafter, interpretation of relationships between the e-learning program and learning responses was performed.

## Results

### Quantitative data

In total, 84 students were included, and 62 (30 control group and 32 e-learning program) completed the six-week e-learning program. Dropout was caused by a lack of motivation and a high workload in the clerkship on top of other commitments.

In the pre-test, no statistically significant difference was present between the control group (M=40.27, SD=6.46) and e-learning group (M= 41.63, SD= 8.19). There was a significant difference in the post-test scores for control (M=51.4, SD=6.43) and e-learning group (M=73.09, SD=5.12) conditions; t(60)=-14.75, p<0.000), suggesting that the e-learning program had a significant effect on the acquisition of knowledge in dermatology (Table 1).

**Table 1:** Pre-test and Post-test scores of fourth-year medical students in the control (n=30) and e-learning group (n=32), at Maastricht University, The Netherlands, 2016-2017

Groups	Pre-test % (SD)	Post-test %(SD)
Control Group	40.27 (6.46)	51.40 (6.43)
E-learning Group	41.63 (8.19)	73.09 (5.12)

KEY: %: Average score of correct answers; SD: Standard Deviation

**Table 2:** Main themes and correlated subthemes concerning students’ perceptions of e-learning in dermatology at Maastricht University, The Netherlands, 2016-2017

Use of the e-learning program		
Learning value	Incentive	Demand for knowledge
		Unfamiliarity/prior knowledge
		Dermatology in curriculum
		Awareness of complexity in the field of dermatology
		Exams/ assessments: Computerized Clinical Cases test/Progress test
		Encountered in other areas
	Agency	Availability
		Not on the work floor
		Access
		Instrumental support
	Pure-learning effects	Initiating learning
		Using books
		Diversity of diseases
		Fits with practice
		Information-text
		Anamneses specifically in dermatology
		Steps as dermatologist
		Visual images
		Questions
		Creation of differential diagnosis
	Post-learning effects	Use of references/websites
		Self-direction of learning- Analyse information
		Self-direction of learning- Structure information
		Collaboration with medical professionals
		Collaboration with students
		Achievement goal orientation
		Recognition in practice
		Use app to check program in practice
		Memorization in practice
		No/need for clerkship

### Qualitative data

The emergent themes and sub-themes that were derived from template analysis are presented in Table 2. There were two primary themes: use of e-learning, and the impact of the program on learning approaches. For each theme, we present clarifying quotes in the following paragraphs.

### Use of the e-learning program

#### Incentive

Regarding the underlying motivation for using the e-learning program, all participants used the program for gaining more knowledge in dermatological topics. Generally, the majority of students agreed that unfamiliarity with, and limited instruction in dermatology during the course of their medical studies encouraged the use of the program (quote 1, Table 3).

This concern could be linked to the common awareness of the relevance of dermatology for various aspects of their future careers in all medical disciplines, as well as encountering questions on dermatological topics in formal assessments and seeing patients with dermatological problems in the clerkships (quote 2, Table 3).

#### Agency

The e-learning program increased students’ sense of agency;

 they could study and learn autonomously, independent of the input from supervisors or the medical training program. Preparations for clerkships, assessments, and social activities were interfering factors for daily use of the e-learning program. Students preferred to work on the e-learning program at home, during the evenings, or at weekends (quote 3, Table 3).

A few barriers of the e-learning program were related to technological issues, e.g., the sharpness of pictures (quote 4, Table 3).

#### Learning value

In this section, we present pure - and post-learning effects. of the e-learning program on students’ learning processes.^20^ Pure-learning effects entail learning during the use of the e-learning program, post-learning effects entail the impact on learning following use of the e-learning program.^20^

#### Pure-learning effects

The interviewees mentioned specific features where the material of the e-learning program had additional learning value

for the diseases and procedures in dermatology. The selected cases and associated questions enabled students to get acquainted to the specific steps in a dermatological anamnesis, identifying skin disorders through images, and formulating a differential diagnosis (quote 5, Table 3).

Students stated that the pre-selected questions specifically guided a deeper understanding of the cases. Most of these cases included diseases that were also encountered in practice. In this way, as stated by the students, the controlled introduction of knowledge via the cases allowed students to focus on a specific subject, as opposed to being overwhelmed by information in dermatological textbooks (quote 6, Table 3).

The field of dermatology was perceived as challenging as it represents a large number of possible diseases, and requires detailed knowledge to formulate a differential diagnosis.^1^^,^^2^ Students were required to compare images of skin diseases and to link these with accurate anamnestic information. Therefore, the combination of visual images and patient history in the cases in the e-learning program facilitated the recognition of diseases, allowing students to search for differences between diseases when presented with similar features. In addition, the multiple-choice questions were seen as helpful, as the possible answers each represented a differential diagnosis. This was perceived as helpful for the formulation of a differential diagnosis (quote 8, Table 3).

#### Post-learning effects

Regarding the post-learning effect of the program, students noted that the links to websites initiated the use of the references and the creation of documents for personal learning. In addition, use of the program stimulated discussion of information with medical professionals and other students and aided in the recognition of skin problems in the clinic.

The references helped to review wrong answers in a purposeful way. Feedback on the answers was an important feature for the self-evaluation of their knowledge (quote 9-10, Table 3). Links to websites were also used to further study the mechanisms of diseases. Most students kept a document as an ‘overview’ for themselves, leading to improved retention of knowledge (quote 11, Table 3).

Some students discussed the cases in the program with colleagues or medical professionals in the workplace. By discussing cases, more in-depth information was gained, improving the insights in the disease (quote 12, Table 3). Use of the program also encouraged students to exchange information and discuss mechanisms; this was considered helpful given the unfamiliarity with dermatological diseases, and the desire to do well in assessments (quote 13, Table 3).

Recognition of diseases in the clerkship facilitated students’ learning by forming a more coherent picture of the patient and disease. Some students used the encounter with patients to return to the program, allowing them to consolidate their knowledge (quote 14, Table 3).

## Discussion

An e-learning program in dermatology on smartphones offers a promising approach to enhance students’ knowledge and learning approaches. Our results resonated with recent studies on the use of e-learning in dermatology.^1^^,^^3^^,^^7^^,^^11^

**Table 3:** Quotes 1-14 of interviews 1-9 concerning students’ perceptions of e-learning in dermatology at Maastricht University, The Netherlands, 2016-2017

Quote	
1	*“I was really pleased that this app was offered as I was afraid I would have had limited knowledge on these topics. Now I did get assigned a workplace in the dermatology department during the clerkship, but imagine that this had not been the case, I would say the app is very useful for gathering basic knowledge in this field. It's like I said, there is very little attention for this discipline in the pre-clinical phase of the undergraduate training. These cases in the app are just very accessible in terms of questions and time you need to spend on it”. *(Interview 6, male, age 22 years)
2	*“I do not know yet what discipline I would like to do [in post-graduate training], but I do know that skin disorders are frequently seen among patients. Perhaps I want to be a general practitioner (Family medicine), and then I would see all kinds of patients with skin conditions. Through the app, I could boost my knowledge in dermatology, because I knew I would not have a workplace in the dermatology department during the clerkship or more lectures on these topics”. *(Interview 5, male, age 21 years)
3	*“Often, I used the e-learning program during the evening, and I did a couple of cases at once. I have not really tracked how long I worked on it. By just reading the cases at my own pace, I tried to learn”. *(Interview 3, female, age 21 years)
4	*“The quality was fine, although sometimes pictures were not sharp enough. Some images moved when enlarging images on the screen of the mobile phone and then I got stuck in the whole process”. *(Interview 1, female, age 21 years)
5	*“Questions are asked in a certain order, in steps. You first hear the history of the patient and then a certain change is introduced, and you get the question: ‘What do you think now?’ Through these questions, you are encouraged to look at the problem from a dermatological perspective. Also, you become more acquainted with the steps that you have to take”. *(Interview 1, female, age 21 years)
6	*"I think…as the cases from the app were constructed in a certain order, this defined what I was going to study, and I was comfortable with that. By using a book including enormous amounts of information and names of diseases, I thought ‘where am I going to start?’ In this application, receiving selected cases just makes you start” I got more structured. Just like when you see a patient in the clinic, and later on you will focus your study on the patient, now you get a case and use that as a focus for study, this works well for my retention of knowledge”. *(Interview 3, female, age 21 years)
7	*"I think the written [text] information and history of the patient is more useful for me because there are also things like 'is it light-sensitive?" Or "did it fade away quickly?" which is more useful for me than a picture. Often, I’m faced with red rash, for example, which can still indicate a lot of different things and be very divergent". *(Interview 2 female, age 22 years)
8	*“If I don’t know the answers, then the presentations of the various diagnoses in the program are quite practical. If you don't know the answer based on images, you are forced to think beyond that and go through several options. We don’t have that much knowledge to actually construct differential diagnosis ourselves. Otherwise, we have to go over all skin disorders”. *(Interview 7, female, age 21 years)
9	*“I used the program especially when I gave the wrong answer on a question, and did not understand why. Then I used the link to check the website”. *(Interview 2, female, age 22 years)
10	*“It's a bit like the progress test [=longitudinal knowledge test], I think, you might know what the answer is, but if you do not know why you remain on the same level. So, you want to simply sort out the other options”. *(Interview 9, male, age 21 years)
11	*“I kept a document for myself in which I underlined the headlines of cases and questions, and added information from the external websites. Then I remembered it better because I could link the information together with the case and its question”. *(Interview 4, female, age 22 years)
12	*“Often I had some time to work on the cases and questions during my clerkship. There were also several people I could ask for help, like the residents from the internal medicine department. Today I asked someone for the question on the diagnosis in the case of lupus, which was great”. *(Interview 5, male, age 21 years)
13	*"I noticed that many students do not really know much about dermatology and that such an app would provide an effective solution in this situation, especially since those questions always come back in the progress tests. I also noticed this when we were discussing these questions; then they would say 'oh, okay, now I know I see how it works, now I get it”. *(Interview 6, male, age 22 years)
14	*“When I encounter this disease in practice, then it hits me, oh wait, I recognize this, for example in the case with the seborrheic keratosis and basaliomas. These are images that are very common, and sometimes this type of seborrheic keratosis is also not quite "typical" as it looks. So, when I saw it in real-life, I had to doubt it, but then with the use of the app, I realized that I still noticed features that could be seen in images that I did not see in practice. So, I used this to switch from one to another”. *(Interview 1, female, age 21 years)

Wahlgren and colleagues described several benefits of the use of e-learning programs for students, such as the ability to work at their own pace, and allowing repetition and reflection, which is not always possible when working with patients in a clinical setting.^1^ These findings are comparable to the observed effects on learners’ ‘agency’ and the pure-learning effects in the current study. The perception that knowledge was acquired more rapidly was also identified by Wahlgren and colleagues, which might be a result of focusing on a specific subject or topic, as opposed to being overwhelmed by information in dermatological textbooks.

The data in the interview study also pointed to performance- or learning- goal orientation as an important theoretical framework.^21^ A student with a performance- goal orientation is focused on proving competence or ability, and how this is judged in comparison to others; whereas, in a learning-goal orientation, a student is focused on learning and increasing competence for the personal benefit.^21^^,^^22^

Results showed both aspects of the model. Students used the cases with information and feedback from the questions to manage their desired and actual performance and to identify  their learning  needs. In  addition, asking  peers  for feedback or information supported their learning. However, students also indicated that the features of the e-learning program supported their performance improvement, allowing them to perform better in the exam at the end of the clerkship, in addition to improving their progress as learners. Options such as immediate feedback could be provided by this technology, which could identify weak points, so that students could learn more quickly from their knowledge gaps, within the context of both a performance- and learning goal orientation.^7^^, ^^12^

E-learning programs are becoming popular as an additional educational format in medical education.^9^^, ^^12^ However, even if e-learning is appreciated by students, the efficacy of e-learning must be rigorously evaluated and compared to conventional teaching. Studies evaluating online modules in Dermatology showed that students significantly increased their performance level in examinations.^1^^,^^3^^,^^7^^,^^11^In our study, we also showed that the e-learning program significantly increased knowledge acquisition in dermatology when combined with conventional teaching methods. More studies are required to evaluate the impact of e-learning teaching formats used either alone or in conjunction with conventional teaching methods.

We acknowledge several limitations in this study. First, we only evaluated the use of the program in one university. Our results may therefore not be generalizable to other areas and medical curricula. Second, the study focused solely on short-term retention of knowledge. Future work could investigate whether the e-learning program would also be suitable as a teaching method in improving long-term knowledge retention in the subject of dermatology.

## Conclusions

This study indicated that the use of an e-learning program in dermatology by undergraduate medical students in their clinical phase had a positive effect on learning outcomes. Use of the e-learning program led to a significant improvement in basic dermatological knowledge. An important finding was the underlying mechanism of learning, which were related to pure-learning effects, i.e., learning while using the e-learning program; and post-learning effects, i.e., learning through the use of references, to create documents, and discuss information with other medical professionals. The importance of novel educational formats as described in this paper is very likely to move beyond the scope of medical students and field of dermatology. In addition to previous studies that focused on students in their pre-clinical phase, this study identified important learning processes of medical students in their clinical phase.^1^^,^^3^^,^^12^^,^^23^^,^^24^ Furthermore, the e-learning program holds promise for other disciplines, such as family medicine, radiology, and pathology. In view of creating more exposure to dermatology in medical education, we suggest complementing conventional teaching formats with an e-learning program on smartphones to assist in students’ learning of dermatological cases and topics.

### Acknowledgements

We are grateful to all of the medical students who were willing to participate in this pilot as well as providing helpful comments. We thank S. van Mierlo and R. van Amelsfoort of Fontys Hogescholen, The Netherlands, for their support during the program development. Finally, we wish to thank Niels van Berkel, School of Computing and Information Systems, University of Melbourne, and Ella Gough, London, United Kingdom, for their helpful feedback on the draft manuscript. Financial support was provided through the Department of Dermatology, Maastricht University Medical Centre, Maastricht, The Netherlands.

### Conflict of Interest

The authors declare that they have no conflict of interest.

## Supplementary material

Supplementary file 1 Interview guide: perceptions of year four medical students on their learning processes
