# Supplementary material for: Use of e-learning in clinical clerkships: effects on acquisition of dermatological knowledge and learning processes
Source: Int J Med Educ. 2018 Jan 17;9:11–7. doi: 10.5116/ijme.5a47.8ab0 (PMC5834826; doi:10.5116/ijme.5a47.8ab0)
Supplement: Supplementary file 1 — Interview guide: perceptions of year four medical students on their learning processes [file ijme-9-11-S1.pdf]

Appendix

Interview guide: perceptions of year four medical students on their learning processes
(Maastricht University, The Netherlands, 2016-2017)

Expectations in relation to e-learning
- Did the e-learning cover the content you expected? Why or why not? What did it teach you?
- What was your goal when studying with this program?

Structure and Contents e-learning app
- How relevant is the content of the e-learning program to cases in daily practice? How was it relevant to you?
- Did the content give sufficient explanation to the knowledge, skills and concepts presented in lectures?
- Did you use links to external websites? If yes, how did you use them? If no, why not?
- Did the use of case studies and scenarios give you a better understanding of cases in dermatology?
- What parts of the e-learning course have you found the most useful and interesting?

Quizzes
- How relevant were the quizzes and tests in the app?
- How high was the quality of the questions in the quiz?
- Were the questions good practice materials?

Time
- What was the (mean) amount of time you spend on this e-learning program?

Interactivity
- This e-learning course provides opportunities for interactive learning. Agree or disagree?

Overall experience
- Can you identify three key concepts or ideas you have learned in this e-learning program?
